# Neuron pruning in temporal domain for energy efficient SNN processor design

**DOI:** 10.3389/fnins.2023.1285914

**Published:** 2023-11-30

**Authors:** Dongwoo Lew, Hoyoung Tang, Jongsun Park

**Affiliations:** School of Electrical Engineering, Korea University, Seoul, Republic of Korea

**Keywords:** spiking neural network, approximation, computation reduction, input-dependent neuron pruning, neuromorphic

## Abstract

Recently, the accuracy of spike neural network (SNN) has been significantly improved by deploying convolutional neural networks (CNN) and their parameters to SNN. The deep convolutional SNNs, however, suffer from large amounts of computations, which is the major bottleneck for energy efficient SNN processor design. In this paper, we present an input-dependent computation reduction approach, where relatively unimportant neurons are identified and pruned without seriously sacrificing the accuracies. Specifically, a neuron pruning in temporal domain is proposed that prunes less important neurons and skips its future operations based on the layer-wise pruning thresholds of membrane voltages. To find the pruning thresholds, two pruning threshold search algorithms are presented that can efficiently trade-off accuracy and computational complexity with a given computation reduction ratio. The proposed neuron pruning scheme has been implemented using 65 nm CMOS process. The SNN processor achieves a 57% energy reduction and a 2.68× speed up, with up to 0.82% accuracy loss and 7.3% area overhead for CIFAR-10 dataset.

## 1 Introduction

Convolutional neural networks (CNN) such as GoogLeNet (Szegedy et al., 2015) and VGG-16 (Simonyan and Zisserman, 2014) have been achieving record-breaking classification accuracies in computer vision benchmarks like CIFAR-10 (Krizhevsky, 2009) and ImageNet (Russakovsky et al., 2015). To achieve state-of-the-art accuracy, deeper and larger neural network architectures with considerable computational costs are required, which is a large burden for hardware implementation. Recently, with a need for running deep neural networks on mobile applications under limited power budget, new computational paradigms have been actively researched (Esser et al., 2016; Rueckauer et al., 2017).

Unlike other classes of artificial neural networks, spiking neural networks (SNNs) perform neural computations using spikes in an event-driven fashion. SNNs use sparse temporal–spatial patterns of spikes to convey information. With event-driven temporal data processing, SNNs are expected to be implemented with energy efficient hardware. IBM’s TrueNorth (Merolla et al., 2014) and Intel’s Loihi (Davies et al., 2018) are the typical examples of energy efficient SNN hardware, where millions of neurons are implemented with a few hundred mW of power dissipation. In terms of functional accuracies, new training methods (Diehl et al., 2015; Rueckauer et al., 2017; Sengupta et al., 2019; Li et al., 2021) are proposed to improve the accuracies of SNNs, where CNN architectures and the parameters are deployed to SNNs. According to Rueckauer et al. (2017), the accuracies of the convolutional SNNs have reached to those of CNNs in computer vision benchmarks such as CIFAR-10 and ImageNet. In spite of the accuracy improvements, since only small portions of neurons are updated in each timestep in SNN, the number of computations at each timestep is much lower than those of CNN. However, in order to get high recognition accuracies, large number of timesteps are still needed, which incur redundant computations with latency overheads.

To reduce the amount of computations, efficient conversion methods from deep CNN to SNN have been proposed (Diehl et al., 2015; Rueckauer et al., 2017; Sengupta et al., 2019; Li et al., 2021). By re-scaling the pre-trained parameters of CNN, a large number of spike-driven computations are reduced. However, as the importance differences among the computations are not considered, all the computations in SNN are processed with equal efforts and the complexity reduction is quite limited. An input-dependent approximate computing approach (Sen et al., 2017) is also proposed for SNN, where less important spiking neurons are skipped for each input spike train. With the inherent error resiliency of neural network, large portion of computations can be skipped. But, the approach suffers from large control and computation overheads, which weakens the effect of the energy reduction gained from the approximate computing.

In this paper, we present an input-dependent computational complexity reduction approach, where temporal domain information is efficiently exploited to remove the computational redundancies that inherently exist in convolutional SNN. In the proposed scheme, the relatively less important neurons are first identified, and those are removed in the temporal domain by monitoring the changes of the neuron’s membrane voltages. Since the approach performs the pruning based on the membrane voltages, the overhead in hardware is minor. For the search of each layer’s pruning threshold, the threshold search process is modeled as a graph search problem, and greedy best-first search is used to find the thresholds for a given target computing reduction ratio. In addition, a layer-wise pre-search procedure is also presented to expedite the overall threshold search to automatically find a good starting point of the pruning threshold. The SNN processor that supports the proposed input-dependent computational reduction technique, has been implemented using 65 nm CMOS process. The implementation results show that the SNN processor shows significant energy reduction with minor hardware overhead.

The rest of the paper is organized as follows. In Section 2, the preliminaries for SNN architectures and the previous computation reduction approaches are introduced. The proposed neuron pruning scheme is presented in Section 3, and the experimental and hardware implementation results are presented in Section 4 and 5, respectively. Finally, conclusions are drawn in Section 6.

## 2 Preliminaries

### 2.1 Spiking neural networks (SNN)

**Figure 1:**
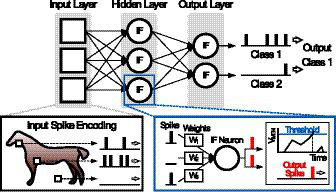
Image classification tasks in Spiking Neural Network (SNN).

Figure 1 shows a typical example of an image classification task in SNNs. From input pixel data, Poisson-distributed spike train is generated with the rate proportional to pixel intensity, and the input spikes are first fed into integrate-and-fire (IF) neurons. The IF neurons integrate synaptic weights of incoming input spikes to its membrane voltage (Vmem) during each timestep of spike train. When Vmem exceeds a predefined threshold voltage (Vth), the neuron fires an output spike to the next layer, and simultaneously its Vmem resets. In the last layer, there are same number of output neurons as the number of output classes. SNN performs the classification task that selects the output class neuron that has maximum spike rate. In order to increase the accuracies of the classification tasks, previous research works (Diehl et al., 2015; Rueckauer et al., 2017; Sengupta et al., 2019; Li et al., 2021) try to convert and deploy the trained weights of CNN to SNN. By employing the CNN architecture and matching the spike rates in SNN to the activation values, SNN have achieved near-lossless accuracies over those of CNN’s (Rueckauer et al., 2017; Sengupta et al., 2019) in various datasets such as CIFAR-10 (Krizhevsky, 2009) and ImageNet (Russakovsky et al., 2015). However, the number of computations, which are needed to catch up the CNN classification accuracy, significantly increases (Diehl et al., 2015; Rueckauer et al., 2017).

### 2.2 Previous computation reduction approaches

Although CNN shows excellent classification accuracies, such performance comes at the cost of an enormous number of computations. To reduce the number of computations, various computation reduction approaches have been studied for CNNs. Zero prediction (Kim et al., 2017, 2018; Akhlaghi et al., 2018) is one of those computation reduction approaches, that aims to predict zeros in the output feature map (ofmap) of CNNs with ReLU. Zero prediction reduces the number of computations by terminating the partial sum computation of the predicted zeros, before the end of the complete computation of a pixel in ofmap. In Akhlaghi et al. (2018), zero prediction is executed by first performing the multiply and accumulate (MAC) operation of the weights having large absolute values, evaluating the partial sum value, and predicting zero based on the evaluated intermediate partial sum. This is possible since a partial sum is sequentially computed in the time domain by a series of MAC operations. In other words, CNNs have temporal domain information in hardware, which has been exploited to reduce the number of computations in Akhlaghi et al. (2018).

Similarly, SNN has inherent temporal domain information in both algorithm and hardware since the information is encoded and processed in a time series of spikes (temporal information in algorithm), which is processed through physical time in an SNN processor (temporal information in hardware). This increase in information in the temporal domain also can cause errors in the network, where inactivated neurons can fire spikes (Li et al., 2022). However, the increase in temporal domain information can also be exploited for computation reductions. In a particular timestep, when a neuron exceeds its threshold value, the membrane voltages (Vmems) of fan-out neurons increase by the weights of the respective connections. As the spike-triggered membrane voltage updates are repeated during the whole timesteps, it incurs a large computational overhead. In this regard, recent conversion methods try to reduce the number of timesteps and decrease the computational overhead. For instance, burst spikes (Li and Zeng, 2022) allows efficient information transmission using spikes in short period of time, and (Bu et al., 2022) proposed activation function that accounts SNN errors during training to greatly reduce the number of timesteps required to achieve comparable accuracies to CNNs. On the other hand, an approximate computing scheme (Sen et al., 2017) is proposed to skip the neuron updates with a minor impact on classification accuracy without modifying the training or conversion of SNN. In this approach, the importance of neurons is obtained based on their output spike rates, and the neuron updates are approximated or skipped for less important neurons. However, the approximate scheme (Sen et al., 2017) needs large additional memories for storing neuron states as well as synapse weight reorganizing process. In order to efficiently take advantage of temporal domain information in SNN, a new computation-skip scheme (neuron pruning scheme) with small overhead is highly needed.

## 3 Neuron pruning in temporal domains (*NPTD*)

In this section, we present an input-dependent computation reduction approach, where the temporal redundancies in SNN are identified and removed with minor accuracy degradation.

### 3.1 SNN training method used in the simulations

Before talking about the pruning techniques, let us describe the training method for SNN used in this work. Among numerous methods to train SNN, ANN-to-SNN conversion method (Diehl et al., 2015; Rueckauer et al., 2017; Sengupta et al., 2019; Li et al., 2021) is selected in this work as it shows classification accuracy comparable to CNNs. A state-of-the-art conversion technique is adopted with Light Pipeline (Li et al., 2021) using the implementation of the authors of the original paper. While the Advanced Pipeline method (Li et al., 2021) can achieve higher classification accuracies, Light Pipeline is chosen as it has a smaller memory overhead when implemented in hardware. Unless otherwise specified, all details regarding the training and conversion process are identical to Li et al. (2021), and the timestep used in the simulations is 128 in the following to explain the proposed techniques.

Although the proposed pruning technique is applied to the above mentioned conversion method as a case example, since temporal redundancies exist in almost any of SNNs, the proposed techniques can also be applied to the SNNs obtained through other training methods.

### 3.2 Overview of neuron pruning in temporal domains (*NPTD*)

**Figure 2:**
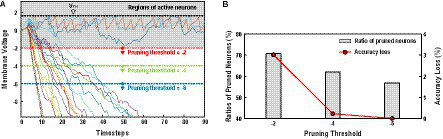
**(A)** Membrane voltages of neurons and three pruning thresholds of case study. **(B)** The ratios of pruned neurons and accuracy losses with three pruning threshold values.

The *Neuron Pruning in Temporal Domain* (*NPTD*) is motivated by the observations that the changes of neuron membrane voltages are predictable. Figure 2A shows the plot of membrane voltages with increasing timestep, which are obtained from the 4th layer of VGG-16 with CIFAR-10 dataset. While monitoring the changes of membrane voltages, two interesting observations are found, which are useful to identify the relatively less important neurons. First, as shown in Figure 2A, unlike the positive membrane voltage values which are reset after firing spikes, the negative membrane voltages of inactive neurons keep decreasing without any reset operations. As the neurons with decreasing membrane voltages are not likely to fire spikes afterward, they do not have any effect on output quality. Those unimportant neurons can be pruned immediately after their membrane voltages reach to a pre-decided threshold values. Second, as presented in Figure 2A, the membrane voltages of the neurons that fire spikes, sometimes go below zero to some degree. Accordingly, if the pruning thresholds are set too high, even the active neurons can be pruned, thus degrading the classification accuracies. As a case study of *Neuron Pruning in Temporal Domain* (*NPTD*), *NPTD* with three pruning thresholds (−2, −4, and −6) are simulated, and the results are shown in Figure 2B. As shown in the figure, when the pruning thresholds are −2, −4, and −6, 70.7, 62.1, and 56.9% of neurons are pruned with 3.02, 0.23, 0.01% accuracy losses, respectively. This means that different pruning thresholds lead to different points of accuracy losses and computation reductions. As the threshold is getting smaller (usually negative values), relatively smaller number of neurons are pruned with less accuracy loss. Whereas larger threshold results in relatively larger number of pruned neurons with larger accuracy loss. *Therefore*, searching a good pruning threshold is definitely needed to minimize the accuracy loss of the NPTD with a given target computation reduction ratio.

### 3.3 Greedy search algorithm to find *NPTD* threshold

When applying *NPTD*, if the pruning thresholds are individually assigned to all the neurons, search space becomes prohibitively large and the memories to store the pruning thresholds should be large as well. Referred from the previous literatures (Bengio et al., 2006; Lin et al., 2017; Wang et al., 2019), where layer-wise search algorithms are utilized to find optimum design points such as bit-widths of quantization or approximation parameters, pruning thresholds of the *NPTD* are searched per layer in this work.

#### 3.3.1 Definition of the search problem

The pruning threshold search problem can be formulated as follows: “Given the target computation ratio (α), determine the pruning threshold of each layer such that accuracy degradation can be minimized.” As an output of the search, a set of pruning thresholds is determined as $P_{th}=\left\{p_{th,1},p_{th,2},\ldots ,p_{th,L}\right\}$ , where $p_{th,n}$ denotes the pruning threshold of nth layer and *L* refers to the number of layers. When considering the target computation ratio (α), as a measure of the number of computations, the *synaptic operation* (SOP) (Merolla et al., 2014) is used. The total number of SOPs ( C ), which means the total number of membrane updates of the neurons in a SNN across the timestep *T*, can be described as follows:


(1) $$C=\overset{T}{\underset{t=1}{\sum }}\left(\overset{L}{\underset{l=1}{\sum }}f_{\mathrm{out},l}\times s_{l}\left(t\right)+n_{l}\right)\text{,}$$

where $s_{l}\left(t\right)$ denotes the number of spikes fired in layer l -1 at timestep t , $f_{\mathrm{out},l}$ denotes the number of fan-out synapses from layer l -1 to layer l and $n_{l}$ denotes the number of neurons in layer l and L refers to the number of total layers.

#### 3.3.2 Search procedure

**Figure 3:**
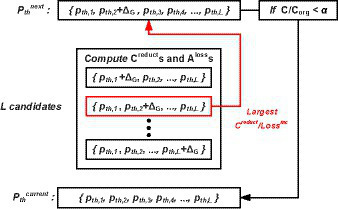
Greedy search procedures to find the set of pruning thresholds Pth.

When brute force search is applied to the search space, the time complexity is as large as O (*n^L^*), where *L* is the number of total layers and *n* refers the number of possible threshold candidates. Considering the prohibitively large time complexity, we adopt the greedy best-first search (Coles and Smith, 2007) in our approach. The conceptual diagram of the Greedy search is presented in Figure 3. The algorithm starts from the initial search point of $P_{th}^{\mathrm{init}}=$ { $p_{th,1}^{\mathrm{init}},p_{th,2}^{\mathrm{init}},\ldots ,p_{th,L}^{\mathrm{init}}$ }, where each of $p_{th,k}^{\mathrm{init}}$ values are very small. Then, we increase $p_{th,k}^{\mathrm{init}}$ by adding $\Delta_{G}$ . After adding $\Delta_{G}$ to each one of *L* candidates independently, we calculate $C_{i}^{\mathrm{reduct}}/Loss_{i}^{inc}$ of *L* cases, where $C_{i}^{\mathrm{reduct}}$ means the amount of computation reduction, and $Loss_{i}^{inc}$ denotes the corresponding *output loss* (cross entropy loss) increment over subset of the training dataset S (an identical S is used during the entire search). Then, we find the one that incur largest $C_{i}^{\mathrm{reduct}}/Loss_{i}^{inc}$ with pruning threshold increase of $\Delta_{G}$ . This process is repeated until $C/C^{org}$ reaches the target computation ratio α, where $C^{org}$ is the SOP before applying the proposed *NPTD*. While the search problem is to find the pruning thresholds with minimum accuracy loss at a given target computation ratio, the *output loss* is used instead of accuracy during the search. It is because *output loss* and the classification accuracy of the network are closely related, *output loss* is widely used instead of accuracy in previous works (Shih and Chang, 2020; Gholami et al., 2021). The pseudocode of our greedy best-first search is presented in Algorithm 1.

**Algorithm 1:**
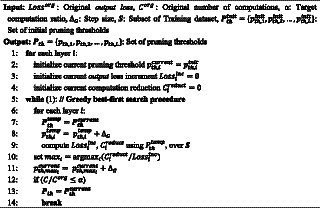
The greedy best-first search to find threshold Pth.

### 3.4 Layer-wise pre-search procedure

**Figure 4:**
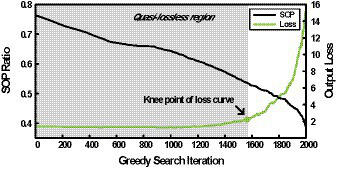
SOP ratio and output loss with respect to the number of iterations when the greedy best-first search is performed.

Although the greedy best-first search algorithm can find a set of pruning thresholds for *NPTD* while providing efficient trade-offs between accuracies and computational complexity, we still have room for improvement in terms of the runtime of the search. This can be observed in Figure 4, where the plot of SOP ratio and *output loss* with respect to the number of iterations of the greedy search (i.e., loop iteration of line 5 in Algorithm 1) is shown when the initial pruning thresholds of all the layers are set to −20 for VGG-16 with CIFAR-100. The SOP ratio is computed by dividing the current SOP by the SOP without *NPTD* applied. We can notice from Figure 4 that SOP ratio is decreasing during the whole search, however, *output loss* shows very little change before the knee point of the curve. This means that a large number of the iterations are performed while *output loss* increases very small, hence this region can be considered as the *quasi-lossless region*.

To reduce the total number of iterations in the greedy search and the time spent in this *quasi-lossless region*, searching the initial threshold of each layer with minor *output loss* change can be considered. To automatically find the set of initial pruning thresholds that can significantly reduce the *quasi-lossless region* with minor change on the *output loss*, we present the layer-wise pre-search based on the bisection method (Burden and Faires, 1985). First, considering the complexity of the search, the pre-search problem can be divided into each of a layer-wise search. A layer-wise approach has been selected, since the error of the output layer, which directly affects the *output loss*, is upper bounded by the weighted linear combination of layer-wise error (Li et al., 2021). In other words, the layer-wise error introduced by the *NPTD* will have a negligible impact on the *output loss* if the layer-wise error introduced by the *NPTD* is small enough. This can be exploited in the layer-wise search, as finding a pruning threshold with minor effect to the *output loss* (i.e., rough threshold with some margin) using a fast layer-wise search will allow the quick search of pruning thresholds of the whole network.

**Figure 5:**
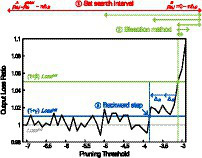
The outline of the pre-search procedure on output loss ratio with respect to pruning threshold. The figure is obtained from the second convolutional layer (conv1_2) of VGG-16 with CIFAR-100.

The outline of the pre-search procedure for a layer is as followings and it is also presented in Figure 5.

Set search interval of the pruning threshold [ $p_{th,i}^{A}$ , $p_{th,i}^{B}$ ] for the bisection method.
Using the bisection method, find a pruning threshold that results in the *output loss* that is close to $\left(1+\beta \right)Loss^{\mathrm{init}}$ , where $Loss^{\mathrm{init}}$ is the *output loss* before the search of this layer.
Perform backward steps until the *output loss* is smaller than $\left(1+\gamma \right)Loss^{\mathrm{init}}$ , by decreasing the pruning threshold found in ② with a step size of $\Delta_{B}$ .

Figure 5 illustrates the pre-search procedure using the plot of *output loss ratio* with respect to the layer-wise pruning threshold. The *output loss ratio* is calculated by dividing the current *output loss* by the $Loss^{\mathrm{init}}$ (*output loss* when the pruning threshold of the layer is the initial pruning threshold, which is −32 in this figure).

As shown in Figure 5, starting from the first layer of the network, the step ① first sets the search interval for current layer by starting from [ $P_{th}^{\mathrm{global}}$ , 0 ]. Then, both endpoints are reduced by $\Delta_{B}$ , until the *output loss* of the right endpoint becomes smaller than $\left(1+\beta \right)Loss^{\mathrm{init}}$ . This step is needed since simply setting the search interval to [ $P_{th}^{\mathrm{global}}$ , 0 ] can make the bisection method to fail at the beginning. It is because setting the pruning threshold to 0 makes so many neurons to be pruned, which results in almost no output spike generations, thus making the *output loss* to be a very small. Then, the step ② performs bisection for predefined iterations (*MI*) to find a pruning threshold that has *output loss* close to $\left(1+\beta \right)Loss^{\mathrm{init}}$ . Hyperparameter β is the bisection target ratio and it is added as a margin to ensure stable bisection search. Without β, fluctuation of *output loss* can introduce multiple roots, failing to find a pruning threshold. Lastly, in step ③ of Figure 5, the pruning threshold found in step ② gets decreasing to ensure negligible layer-wise error introduced by *NPTD*. After all three steps, pre-search of the preceding layer is performed. When the layer-wise pre-search is finished, a set of pruning thresholds found is used as the initial pruning threshold set of the greedy best-first search. The detailed procedure is described as pseudocode in Algorithm 2.

**Algorithm 2:**
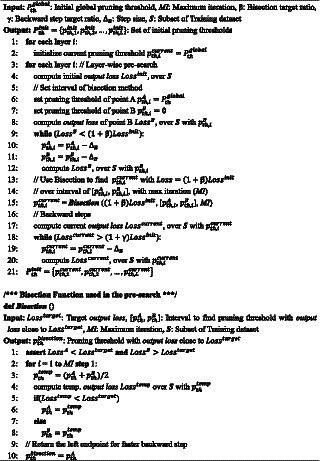
The layer-wise pre-search to find threshold P_th_^init^.

Regarding the hyperparameters used in the pre-search, $P_{th}^{\mathrm{global}}$ should be small enough not to add significant layer-wise error. A good starting point is found to be around −32 or − 64. In the case of *MI*, it should be set with consideration of the search interval and the desired resolution of the threshold pre-search. For instance, when the desired resolution is 1 and $P_{th}^{\mathrm{global}}$ is −32 (= − 2^5^), *MI* of 5 can be used to achieve the desired resolution of 1. In case of β, a value between 0.05 ~ 0.1 is found to be a good balance point between the stability of the search and the total time spent for backward steps. For γ, the value around 0.01 works well for various conditions. It is noteworthy that the value of β has little to no effect to the final results of the pre-search, as long as γ is set to a small value (e.g., 0.01). This is because backward steps diminish the error increment caused by β.

## 4 *NPTD* simulation results

In this section, we present the simulation results of the proposed *NPTD* with threshold search algorithms. The simulations have been performed using PyTorch (Paszke et al., 2019) and the ANN-to-SNN conversion method used is the Light Pipeline proposed in Li et al. (2021). Unless otherwise specified, hyperparameters used in the simulations are as followings: $\alpha =0.5,\Delta_{G}=0.1$ and the number of elements in S is 1,024 for the greedy best-first search. For the layer-wise pre-search, $P_{th}^{\mathrm{global}}=-64,MI=6,\beta =0.05,\gamma =0.01,\Delta_{B}=1.0$ and the number of images in S is 2048. The number of elements in S is set to a minimum value such that the stability of the threshold search is sustained.

### 4.1 Classification accuracies and SOPs

Table 1 shows the simulation results of the classification accuracies and the number of SOPs for VGG-16 and ResNet-20 on CIFAR-10 and CIFAR-100. Considering the baseline accuracy of the networks, results with timesteps over 32 are reported. In Table 1, the values in the parenthesis of accuracy and SOP are the accuracy drop compared to the baseline accuracy and the ratio of the SOP compared to the baseline SOP, respectively. Overall, the layer-wise pre-search finds pruning thresholds with SOP ratio within the range of 0.5 ~ 0.7 and the SOP reduction ratio is generally higher when the timestep becomes larger. The accuracy drops are less than 0.6% for all cases. Interestingly, for the case of *T* = 128 and 256 for VGG-16 on CIFAR-100, accuracies increase compared to the baseline.

**Table 1:** Classification accuracies and SOP simulation results.

VGG-16, CIFAR-10								
	*T* = 32		*T* = 64		*T* = 128		*T* = 256	
	Accuracy	SOP	Accuracy	SOP	Accuracy	SOP	Accuracy	SOP
Baseline	93.49	2.42E+8	94.97	4.98E+8	95.28	1.02E+9	95.55	2.05E+9
PS	93.49 (0.00)	1.73E+8 (0.71)	94.68 (0.29)	2.78E+8 (0.55)	95.09 (0.20)	5.33E+8 (0.52)	95.03 (0.52)	8.40E+8(0.41)
PS + GS	91.76 (1.73)	1.20E+8 (0.49)	94.05 (0.92)	2.48E+8 (0.49)	94.99 (0.29)	5.03E+8 (0.49)	-	-

ResNet-20, CIFAR-10								
	*T* = 32		*T* = 64		*T* = 128		*T* = 256	
	Accuracy	SOP	Accuracy	SOP	Accuracy	SOP	Accuracy	SOP
Baseline	94.51	2.71E+8	95.19	5.48E+8	95.52	1.11E+9	95.59	2.23E+9
PS	94.47 (0.04)	1.96E+8 (0.72)	95.04 (0.15)	3.65E+8 (0.66)	95.20 (0.32)	6.70E+8 (0.60)	95.06 (0.53)	1.18E+9 (0.52)
PS + GS	93.08 (1.43)	1.36E+8 (0.50)	93.38 (1.81)	2.81E+8 (0.51)	94.96 (0.56)	5.30E+8 (0.47)	95.05 (0.54)	1.14E+9 (0.51)

VGG-16, CIFAR-100								
	*T* = 32		*T* = 64		*T* = 128		*T* = 256	
	Accuracy	SOP	Accuracy	SOP	Accuracy	SOP	Accuracy	SOP
Baseline	59.86	3.78E+8	71.10	7.70E+8	75.15	1.56E+9	77.23	3.15E+9
PS	59.78 (0.08)	2.39E+8 (0.63)	71.10 (0.00)	4.80E+8 (0.62)	75.28(−0.13)	9.14E+8 (0.58)	77.34(−0.11)	1.68E+9 (0.53)
PS + GS	55.78 (4.08)	1.89E+8 (0.50)	69.50 (1.60)	3.85E+8 (0.50)	74.23 (0.92)	7.83E+8 (0.50)	77.21 (0.02)	1.58E+9 (0.50)

ResNet-20, CIFAR-100								
	*T* = 32		*T* = 64		*T* = 128		*T* = 256	
	Accuracy	SOP	Accuracy	SOP	Accuracy	SOP	Accuracy	SOP
Baseline	73.18	3.92E+8	75.52	7.94E+8	76.55	1.60E+9	76.91	3.21E+9
PS	72.75 (0.43)	2.90E+8 (0.74)	75.32 (0.20)	5.35E+8 (0.67)	76.22 (0.33)	9.40E+8 (0.58)	76.39 (0.52)	1.68E+9 (0.52)
PS + GS	68.91 (4.27)	1.97E+8 (0.50)	73.09 (2.43)	3.97E+8 (0.50)	75.96 (0.59)	8.01E+8 (0.50)	76.18 (0.73)	1.60E+9 (0.49)

Baseline, NPTD not applied; PS, layer-wise pre-search; GS, greedy best-first search.

After the layer-wise pre-search, pruning thresholds found by the pre-search are used as the initial thresholds of the greedy best-first search. Since the target SOP ratio α is set to 0.5, most of the search finishes with a final SOP ratio of 0.5 with SOP ratio error of around 0.01. It is also very interesting that the accuracy loss of PS + GS is generally smaller when the timestep of the networks is larger, which is because the model capacity increases as a larger timestep is used for the network. The increase of model capacity is upheld by the higher baseline accuracy as the timestep gets larger. One notable exception in Table 1 is the case of *T* = 256 for VGG-16 on CIFAR-10, which shows an SOP ratio of 0.41 right after the layer-wise pre-search (all simulations for this case consistently show an SOP ratio less than 0.5). For this case, the greedy best-first search is not performed as the target SOP ratio is set to 0.5.

### 4.2 Analysis on the effects of the layer-wise pre-search

In this section, to demonstrate the effectiveness of the layer-wise pre-search (PS), analyses on the search runtime, classification accuracy, and SOP are performed. While the simulations are conducted with parameters explained at the beginning of this section, ‘GS’ in Table 2 is conducted with initial pruning thresholds of 15 for all layers. The reported runtimes are simulated on a single Nvidia TITAN RTX and the results are presented in Table 2. The search runtime of PS + GS is presented in form of ‘(PS runtime) + (GS runtime)’. The application of the proposed layer-wise pre-search has dramatically reduced the runtime of greedy search to 31.4 and 30.4% for VGG-16 and ResNet-20, respectively, while only adding 1.5 and 1.0% of pre-search time overhead for VGG-16 and ResNet-20, respectively. The pruning thresholds obtained by PS + GS also show similar accuracies and SOP reductions compared to GS, showing the effectiveness of the proposed PS.

**Table 2:** The Effects of the layer-wise pre-search on search time and accuracies.

VGG-16, CIFAR-100, *T* = 128			
	Accuracy	SOP	Search runtime
Baseline	75.15	1.56E+9	-
PS + GS	74.23 (0.92)	7.83E+8 (0.50)	1.3 + 25.8 h
GS	73.77 (1.38)	7.82E+8 (0.50)	82.1 h
ResNet-20, CIFAR-100, *T* = 128			
	Accuracy	SOP	Search runtime
Baseline	76.55	1.60E+9	-
PS + GS	75.96 (0.59)	8.01E+8 (0.50)	1.5 + 45.5 h
GS	76.14 (0.40)	8.01E+8 (0.50)	149.4 h

Baseline, NPTD not applied; PS, layer-wise pre-search; GS, greedy best-first search.

**Figure 6:**
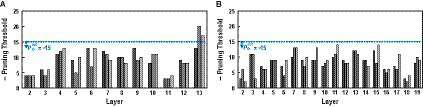
The examples of the pruning thresholds found by the layer-wise pre-search from **(A)** VGG16 and **(B)** ResNet-20. Bars with identical color are the thresholds from the identical network [i.e., black bars in **(A)** are pruning thresholds searched from VGG-16 of an identical random seed].

Please note that for VGG-16, using both the pre-search and the greedy search shows higher accuracy than only using the greedy search, and the reason for this outcome is shown in Figure 6A, where the pruning thresholds found by the layer-wise pre-search are shown for VGG-16 on CIFAR-100. Before explaining further, please recall that the layer-wise pre-search finds the pruning threshold on the left side of the knee point of the loss curve as explained in Figure 5 (i.e., near the end of the quasi-lossless region). In Figure 6A, layer 13 has a smaller pruning threshold found by the pre-search than −15. Such outlier layer makes the greedy search start from a pruning threshold on the right side of the knee point, and it causes the classification accuracy to deteriorate from the beginning of the greedy search. On the other hand, for the ResNet-20 as shown in Figure 6B, the pruning threshold found by pre-search is below −15 for all the layers, and using only the greedy search shows slightly higher accuracy.

## 5 Hardware implementation

This section presents the SNN processor that implements the proposed neuron pruning techniques. The baseline architecture is designed based on Sen et al. (2017), and the proposed *NPTD* has been added to the baseline. 65 nm CMOS standard cell library has been used for the implementation, and the energy results are obtained from post-layout simulations using the CIFAR-10 dataset. Considering the required on-chip memory size to implement the network to hardware, a small convolutional SNN is selected for hardware implementation. The network has 3 convolutional layers and 1 fully connected layer, arranged as architecture of 48c5-AP2-96c5-AP2-96c5-AP2-10. In the network architecture, 48c5 means a convolutional layer with 48 5 × 5 convolutional filters, AP2 means 2 × 2 average pooling layer, and 10 means fully connected layer with 10 neurons. In the case of the first convolutional layer, it is processed off-chip due to the direct input encoding used in Li et al. (2021). The hyperparameter used for the pruning threshold search is the same as the ones specified in section IV, with exception of α=0.3 . The network shows 88.02 and 87.2% accuracy without and with the proposed *NPTD*, respectively, on CIFAR-10 with 128 timestep.

### 5.1 Overall hardware architecture

**Figure 7:**
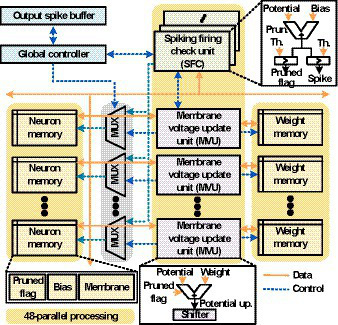
Block diagram of the SNN processor.

Figure 7 shows the block diagram of the proposed SNN processor which consists of an array of 48 spike firing check (SFC) units and an array of 48 membrane voltage update (MVU) units. It also contains a set of neuron memories that are membrane voltage memories, bias memories, and pruned flag memories. The synaptic weights are stored in weight memories, and the global controller orchestrates the overall operations. In the proposed architecture, when a spike is generated, all the membrane voltages of its fan-out neurons are updated, and 48 MVU operations are performed in parallel considering the channel sizes of the kernels (48 and 96). For example, in case of Conv1 (48 × 5 × 5c) layer, each neuron has 48 × 5 × 5 fan-out neurons. So, when a spike is generated from Conv1 layer, 48-parallel MVU computations are performed 25 times to update 48 × 5 × 5 fan-out neurons. To support 48-parallel processing, 2 × 48 banks of membrane voltage memories are accessed to simultaneously read and write the membrane voltages of 48 neurons. Here, half of the memory banks read the membrane voltages of 48 neurons, and the other half memory banks write the membrane voltages that are previously processed. Since the bias memories and weight memories perform only read operation per each neuron, doubling the number of banks is not necessary to support 48-parallel processing. So, 48 banks are used in the architecture for the bias memories and weight memories.

**Figure 8:**
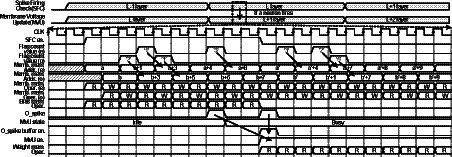
Timing diagram of the proposed SNN processor.

The timing diagram of the proposed architecture is shown in Figure 8. First, SFC array loops over neurons in a certain layer L, and it adds bias values to membrane voltages. In addition, SFC unit checks if the membrane voltages are above Vth. If not, it moves on to the next neurons in the layer L. When neurons fire output spikes, MVU unit array is activated, and it reads the membrane voltages and the synaptic weights for the fan-out neurons in the layer L + 1. As previously described, 2 banks of membrane voltage memories are used as a pair of ping-pong buffers because write operations are also needed to update the membrane voltage values. So, each neuron is updated within one clock cycle. If MVU units are busy when output spike is fired, the indices of output spikes are stored in the output spike buffer and the index is delivered to MVU unit when MVU units are ready. After updating all the fan-out neurons of the output spikes stored in spike buffer, SFC array resumes the operations over the neurons in the layer L. When all the neurons in the layer L are processed, the SFC unit moves to the next layer L + 1. In the procedure, flag count signals inform how many neurons can be skipped at once from the current processing neuron, and using the flag count signal, neuron indices and the corresponding addresses of memories are generated to skip the computations.

In the proposed SNN processor, 11 bits are used to store the membrane voltages with a negligible accuracy loss of 0.05% compared to the floating-point baseline. However, larger bit-widths are needed to implement *NPTD* because the pruning threshold *P_th_* can be larger than the threshold voltage (Vth). In other words, the proposed *NPTD* needs wide dynamic ranges for the negative membrane voltages to represent values down to the pruning thresholds. Since large dynamic ranges are only needed for negative values, using additional bits to represent both positive and negative membrane voltages can be wasteful. So, dynamic fixed-point arithmetic is employed to allow a wider dynamic range for the negative values to realize *NPTD* without adding too much hardware overhead. For example, when the magnitude of the *P_th_* of a layer is 5 times of Vth, 3 bits $\left(=\log_{2}\left(V_{th}/P_{th}\right))\right)$ are shifted when representing the negative values, and when the membrane voltage becomes a positive value, the number representation changes back to normal. The dynamic fixed-point allows a simple yet efficient implementation of the *NPTD* without accuracy loss and additional memory overhead due to increasing bit-widths.

In the proposed architecture, although power consumption can be improved with the neuron pruning by simply not performing the computations of pruned neurons, it cannot increase the throughput of the processor. So, to increase the throughput of the proposed SNN processor, the pruned neurons are identified ahead by counting stored flags using leading one (zero) counter (Miao and Li, 2017) and computations of the identified neurons are skipped.

### 5.2 Hardware implementation results

**Figure 9:**
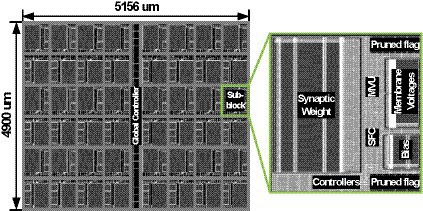
The layout of the proposed SNN processor.

**Figure 10:**
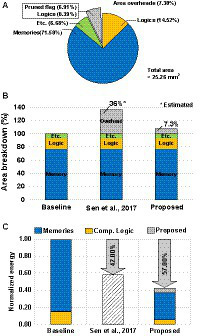
**(A)** Area breakdowns of the proposed SNN processor. **(B)** Area comparison with the previous input-dependent pruning scheme. **(C)** Normalized energy benefits with CIFAR-10 dataset.

The proposed SNN processor has been Verilog coded, and it is synthesized using Synopsys Design Compiler with 65 nm CMOS standard cell library. The netlists are placed and routed using Synopsys IC Compiler, and Figure 9 shows the layout of the SNN processor and its sub-block module. The processor occupies 5,156 × 4,900 um2 of area, and it consists of 48 sub-blocks and a global controller. Each of sub-block has one synaptic weight memory, two banks of membrane voltage memories, eight banks of pruned flag memories, one bias memory, and MVU and SFC units. Figure 10A shows the area breakdown of the SNN processor. Approximately 71% of the area is occupied by the memories storing synapse weights, membrane voltages, and bias values. In addition, the proposed *NPTD* incurs 7.3% area overhead to the baseline architecture. The previous input-dependent pruning scheme (Sen et al., 2017) needs approximately 35% overhead to implement the pruning scheme, which mainly consists of memory to store the target neuron index of each synapse based on our redesigned implementation of (Sen et al., 2017). On the other hand, the proposed *NPTD* needs the memories only for storing 1-bit flag per each neuron instead of the indices of target neurons (Sen et al., 2017). Therefore, the memory area overhead to implement the input-dependent pruning scheme is significantly reduced for the proposed *NPTD*. As presented in Figure 10B, the area overhead for skipping computations is around 7.3%.

When processing images from the CIFAR-10 dataset, the proposed SNN processor can process 1,531 classification tasks (frames) per second by consuming 109.1uJ for each frame. Figure 10C shows the energy reductions of the proposed *NPTD* with the CIFAR-10 dataset. Using the proposed neuron pruning scheme, a significant portion of membrane update computations are skipped, and by skipping the membrane updates of the pruned neurons, the SNN processor saves 57% of energy with 0.82% accuracy loss. In addition to that, the throughput of the processor increases from 570fps to 1531fps by the proposed *NPTD*.

Table 3 shows the comparisons with other SNN processors (Sen et al., 2017; Deng et al., 2020; Kuang et al., 2021; Lew et al., 2022). The previous input-dependent complexity reduction technique has been proposed and implemented in Sen et al. (2017). But, Sen et al. (2017) has only reported accuracy losses, but not actual accuracies. SNN processors with the flexibility of supporting multi-chip based SNN processing have been implemented by Kuang et al. (2021) and Deng et al. (2020). While these two processors are designed for SNNs, but also support a variant of ternary ANN and report CIFAR-10 accuracies of ternary ANN. In the case of (Lew et al., 2022), it is an SNN processor specifically designed for temporal SNNs with a single spike per neuron, and it uses external DRAM to allow the execution of large SNN models. Thanks to the proposed *NPTD*, the lowest energy per image has been achieved by the proposed SNN processor. In Table 3, to compare the processors implemented using different process technologies, the normalized energy consumptions have been obtained using the following equation (Rabaey et al., 2003):

**Table 3:** Hardware performance comparison.

	Sen et al., 2017 [12]	Kuang et al., 2021 [25]	Deng et al., 2020 [26]	Lew et al., 2022 [27]	This work
Technology	45 nm	65 nm	28 nm	28 nm	65 nm
Supply voltage	-	1.2 V	0.85 V	0.99 V	1.2 V
Frequency	1 GHz	192 MHz	300 MHz	250 MHz	357 MHz
Area	0.34 mm2	107.22 mm2	14.44 mm2	0.9102 mm2	25.26 mm2
Network model	-	8 layers (Ternary ANN)	15 layers (Ternary ANN)	VGG-16 (Temporal SNN)	4 layers (Rate SNN)
Accuracy (CIFAR-10)	-	85.76%	89.5%	91.7%	87.2%
Inference throughput	-	181 fps	46,827 fps	327 fps	1,531 fps
Inference energy (Normalized to 65 nm, 1.2 V)	-	-	129 uJ (1,385 uJ)	327 uJ (2,589 uJ)	109.1 uJ
Measurement	Pre-layout simulation	Chip	Chip	Pre-layout simulation	Post-layout simulation


(2) $$\mathrm{Energ}y_{\mathrm{norm}}=\mathrm{Energy}\times \left(\frac{65}{\mathrm{process}}\right)\times {\left(\frac{1.2}{\mathrm{supply}\;\mathrm{voltage}}\right)}^{2}$$

The normalized energies show even more difference between this work and previous works, which further highlights the energy reduction effects of the proposed *NPTD*.

## 6 Conclusion

In this work, we present a neuron pruning in temporal domain (*NPTD*) approach, an input-dependent neuron pruning technique that efficiently removes temporal redundancies in the convolutional SNNs. The proposed *NPTD* skips less important neuron operations by identifying relatively unimportant neurons, based on the membrane voltage of the neurons and pre-decided pruning thresholds. The pruning thresholds are also searched using the proposed layer-wise pre-search and greedy best-first search algorithms. With the proposed neuron pruning schemes and pruning search algorithms, a target SOP reduction can be reached with good accuracy computation trade-offs. The *NPTD* has also been implemented to the SNN processor using 65 nm CMOS process and the processor achieves 57% energy reductions and 2.68 × speed ups, with 0.82% accuracy loss for CIFAR-10 dataset. The proposed input-dependent neuron pruning technique can assist the use of SNNs in edge devices, particularly for low energy applications with limited hardware resources.

## Data availability statement

The original contributions presented in the study are included in the article/Supplementary material, further inquiries can be directed to the corresponding author.

## Author contributions

DL: Conceptualization, Methodology, Writing – original draft. HT: Conceptualization, Writing – original draft. JP: Writing – review & editing.
