# Supplementary material for: Neuron pruning in temporal domain for energy efficient SNN processor design
Source: Front Neurosci. 2023 Nov 30;17:1285914. doi: 10.3389/fnins.2023.1285914 (PMC10719842; doi:10.3389/fnins.2023.1285914)
Supplement: Supplementary file 1 [file Data_Sheet_1.PDF]

Supplementary Material

Neuron Pruning in Temporal Domain for Energy Efficient SNN Processor Design

Dongwoo Lew[1], Hoyoung Tang[1], Jongsun Park[1*]

[1]School of Electrical Engineering, Korea University, Seoul, South Korea

*** Correspondence:**
Jongsun Park
jongsun@korea.ac.kr

1 NPTD on Spiking Audio Dataset

In this section, the results of NPTD on spiking heidelberg digits (SHD) dataset (Cramer et al., 2022) is presented. SHD dataset is a spiking audio dataset, which consists of approximately 10000 recordings of spoken digits from 0 to 9 in both German and English language. The SHD dataset has 8156 and 2264 samples on the training dataset and test dataset, respectively. For the input pre-processing, spatio-temporal bins were used to reduce the input dimensions (Hammouamri et al., 2023). Input neurons were reduced from 700 to 140 by binning every 5 neurons; for the temporal dimension, 10 ms of discrete timestep is used and zero right-padding in temporal domain is used to match the timestep in a batch. The resulting input dimension is 140 in the spatial domain and around 100 in the temporal domain.

SNN with learned synapse delays (Hammouamri et al., 2023) is adopted for the network architecture and learning algorithm. The SNN has two hidden layers and an output layer, where all layers are fully connected layers with learned synaptic delays. The network is trained using the surrogate gradient method (Neftci et al., 2019) and the details of the training follow (Hammouamri et al., 2023).

SUPPLEMENTARY TABLE 1
RESULTS FOR SHD DATASET

**Two layer fully connected SNN, SHD**			
	Accuracy	SOP	Search runtime
Baseline	95.35	6.81E+8	-
PS	95.30 (0.05)	6.67E+8 (0.98)	30.2 sec
PS+GS	93.47 (1.88)	6.13E+8 (0.90)	30.2+64.4 sec
PS+GS	88.28 (7.02)	5.46E+8 (0.80)	30.2+99.0 sec
PS+GS	81.88 (11.59)	4.80E+8 (0.71)	30.2+123.2 sec

*Baseline: *NPTD* not applied, PS: layer-wise pre-search, GS: greedy best-first searchs

The results for SHD dataset are presented in supplementary table 1, where less efficient between the accuracy and the number of SOP is observed compared to static image datasets, where PS is only able to reduce a small amount of SOP and GS starts to show a more prominent accuracy drop as computation reduction ratio increases. These results are possibly due to the highly dynamic nature of the spike trains in the audio datasets, compared to rate coded spikes from static image datasets such as CIFAR-10/100.

2 Ablation Studies on the Subset Size

In this section, ablation studies to the subset size used in the proposed PS and GS are performed. The accuracy, SOP, and search runtime results with various sizes of subset are presented in Supplementary Table 2 and 3.

SUPPLEMENTARY TABLE 2
LAYER-WISE PRE-SEARCH (PS)
WITH VARIOUS SIZE OF SAMPLED SUBSET

**VGG-16, CIFAR-100, T=128**			
**Subset size**	**Accuracy (drop)**	**SOP (ratio)**	**Search runtime**
Baseline	75.15	1.56E+9	-
512	75.31 (-0.16)	9.34E+8 (0.60)	0.4 hours
1024	75.25 (-0.10)	9.43E+8 (0.60)	0.9 hours
**2048**	**75.28 (-0.13)**	**9.14E+8 (0.59)**	**1.3 hours**
4096	75.13 (0.02)	8.84E+8 (0.57)	3.3 hours

*Baseline: *NPTD* not applied

SUPPLEMENTARY TABLE 3
GREEDY BEST-FIRST SEARCH (PS+GS)
WITH VARIOUS SIZE OF SAMPLED SUBSET

**VGG-16, CIFAR-100, T=128**			
**Subset size**	**Accuracy (drop)**	**SOP (ratio)**	**Search runtime**
Baseline	75.15	1.56E+9	-
256	73.85 (1.30)	7.81E+8 (0.50)	1.3+7.7 hours
512	73.97 (1.18)	7.82E+8 (0.50)	1.3+15.0 hours
**1024**	**74.23 (0.92)**	**7.82E+8 (0.50)**	**1.3+25.8 hours**
2048	74.24 (0.91)	7.82E+8 (0.50)	1.3+59.6 hours

*Baseline: *NPTD* not applied

Results of layer-wise pre-search (PS) with varying subset sizes are presented in Supplementary Table 2 and results of greedy best-first search (PS+GS) are shown in Supplementary Table 3. The search runtimes are measured with a single Nvidia TITAN RTX and the search runtime of PS+GS is presented in form of '(PS runtime)+(GS runtime)'. For PS+GS, pruning thresholds obtained by PS with a subset size of 2048 are used as initial thresholds. The bolded row in the tables means the sample size used to obtain the results presented in the manuscript. For the case of PS, the results show that subset size has negligible effects on the accuracy and SOP, while the search runtime almost linearly increases with respect to the subset size. Similarly, GS shows similar trends of increase in search runtime. However, noticeable accuracy losses are observed when the subset size is smaller than 1024, indicating GS needs careful selection of subset size.

3 References

Cramer, B., Stradmann, Y., Schemmel, J., and Zenke, F. (2022). The Heidelberg Spiking Data Sets for the Systematic Evaluation of Spiking Neural Networks. *IEEE Transactions on Neural Networks and Learning Systems* 33, 2744–2757. doi: 10.1109/TNNLS.2020.3044364.

Hammouamri, I., Khalfaoui-Hassani, I., and Masquelier, T. (2023). Learning Delays in Spiking Neural Networks using Dilated Convolutions with Learnable Spacings. Available at: http://arxiv.org/abs/2306.17670 [Accessed October 22, 2023].

Neftci, E. O., Mostafa, H., and Zenke, F. (2019). Surrogate Gradient Learning in Spiking Neural Networks. doi: 10.48550/arXiv.1901.09948.
